# Saturated Nonsingular Fast Sliding Mode Control for the Crane-Form Pipeline System

**DOI:** 10.3390/e24121800

**Published:** 2022-12-09

**Authors:** Baigeng Wang, Shurong Li

**Affiliations:** School of Artificial Intelligence, Beijing University of Posts and Telecommunications, 100876 Beijing, China

**Keywords:** crane-form pipeline, input saturation, nonsingular fast terminal sliding mode, signum function, chattering

## Abstract

The crane-form pipeline (CFP) system is a kind of petrochemical mechanical equipment composed of multiple rotating joints and rigid pipelines. It is often used to transport chemical fluid products in the factory to tank trucks. In order to realize the automatic alignment of the CFP and the tank mouth, the trajectory tracking control problem of the CFP must be solved. Therefore, a saturated nonsingular fast terminal sliding mode (SNFTSM) algorithm is proposed in this paper. The new sliding mode manifold is constructed by the nonsingular fast terminal sliding mode (NFTSM) manifold, saturation functions and signum functions. Further, according to the sliding mode control algorithm and the dynamic model of the CFP system, the SNFTSM controller is designed. Owing to the existence of saturation functions in the controller, the stability analysis using the Lyapunov equation needs to be discussed in different cases. The results show that the system states can converge to the equilibrium point in finite time no matter where they are on the state’s phase plane. However, due to the existence of signum functions, the control signal will produce chattering. In order to eliminate the chattering problem, the form of the controller is improved by using the boundary layer function. Finally, the control effect of the algorithm is verified by simulation and compared with the NTSM, NFTSM and SNTSM algorithms. From the comparison results, it is obvious that the controller based on the SNFTSM algorithm can effectively reduce the amplitude of the control torque while guaranteeing the fast convergence of the CFP system state error. Specifically, compared with the NFTSM algorithm, the maximum input torque can even be reduced by more than half.

## 1. Introduction

**Figure 1:**
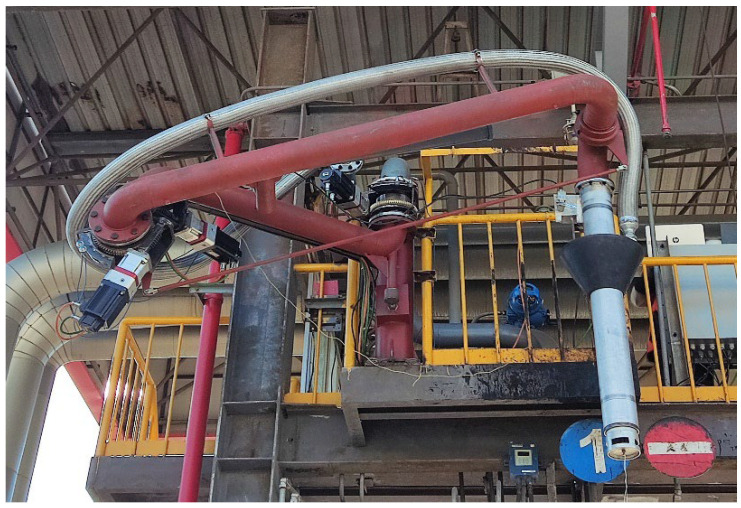
The CFP diagram.

The CFP is a special equipment in the petrochemical industry, which is used for fluid loading and unloading. It uses swivel joints to connect with rigid pipelines and elbows, so as to realize the activity of transferring liquid medium between storage tanks and transportation tank trucks. In the working process, the CFP system is driven by each joint motor, so that the CFP can smoothly extend into the tank mouth of the truck, and ensure that the sealing cover closes over the tank mouth to prevent petrochemical gas overflow during fluid loading and unloading. Figure 1 shows the structure of the CFP. It can be seen that its structure is similar to the industrial manipulator. Therefore, in the automatic alignment system of the CFP, the manipulator control algorithm can be adopted.

In order to solve the control problem of the industrial manipulator, during the past decades, scholars have applied a variety of control algorithms, such as robust PID control [1], backstepping control [2], adaptive control [3], neural network adaptive control [4], fuzzy control [5], active disturbance rejection control [6] and sliding mode control [7].

Among all the above control algorithms, sliding mode control is widely used in the control of various nonlinear systems because of its strong robustness. Early application of the sliding mode control on industrial manipulators can be found in [8], in which the PID sliding mode control algorithm is applied to the controller design of the manipulator. In [9], Yuzheng Guo and Peng-Yung Woo design the classical sliding mode surface and use the fuzzy adaptive algorithm to adjust the controller gain. A robust linear sliding mode controller, which is combined with an adaptive fuzzy logic system, is proposed in [10]. This controller uses the sliding mode control to improve the robustness and control accuracy of the manipulator, and uses the fuzzy logic control to approach various uncertainties and eliminate the chattering. An integral sliding mode control is proposed in [11], which can make the system only have the sliding stage, so as to ensure the robustness and avoid the disadvantage of the approach stage of the linear sliding mode. Further, in [12], Junyoung Lee et al. design an adaptive integral sliding mode control scheme to solve the trajectory tracking control of industrial manipulators with a time-delay. The experiment shows the effectiveness of the proposed algorithm. However, although the controller designed by the above sliding mode control algorithms can guarantee the stability of the system, it can only prove that the tracking error of the system converges to zero asymptotically, which is defective in engineering applications.

For the purpose of a guarantee for the finite time convergence of the system tracking state error, a terminal sliding mode (TSM) algorithm is proposed in [13]. In the TSM control, the control torque will approach infinity in a specific state, resulting in the problem of control singularity. So as to solve this issue, Yong Feng et al. propose a nonsingular terminal sliding mode (NTSM) in [14]. Due to the particularity of its sliding surface, the singular problem can be avoided. Further, reference [15] proposes a NFTSM algorithm, which increases the exponential term of error on the basis of NTSM to improve the convergence speed of the system error when it is far from the equilibrium point. In [16], a second-order fast nonsingular terminal sliding mode controller is designed to achieve the fast convergence of the system state error and ensure the ideal tracking accuracy. In recent years, more and more scholars are paying attention to sliding mode control and putting forward more advanced sliding mode control algorithms. Yuxin Su et al. proposed a new fixed-time sliding surface, and designed a robust control to ensure global approximate fixed time convergence in [17]. An event-triggered sliding mode control method is proposed in [18]. In this method, a sliding mode control law is proposed, and an input event trigger mechanism is introduced to determine when the control signal is transmitted to the actuator side through the network. Reference [19] introduces a practical sliding mode using a state-dependent intermittent control strategy, which drives the system trajectory to the predesigned actual sliding mode band and establishes the actual sliding mode. Its remarkable feature is that it turns off the control when it is unnecessary, which will greatly save control energy and other resources. Although the above sliding mode algorithms can ensure that the system tracking error converges to zero in a finite time, when using the sliding mode algorithm to design the controller, the control torque of the controller may exceed the limit of the actuator [20], and it is difficult to achieve the ideal control effect in projects.

In order to solve this problem, scholars engaged in sliding mode control have carried out the control algorithm for a long time and achieved a lot of results. Shihong Ding et al. propose a SNTSM control for nonlinear second-order systems with input saturation in [21]. This method improves the sliding surface of NTSM so that the controller has an upper bound. In [22], a fast terminal sliding mode control is introduced to solve the nonlinear system with input saturation. In [23], Jun Guo et al. propose an integral sliding mode control algorithm based on saturation functions to solve the bounded input problem. Using the integral sliding mode surface, two robust saturation controllers are designed for nonlinear systems with external disturbances. Reference [24] proposes a new method to maintain nonlinear system stability under disturbance and control saturation; specifically, a new auxiliary state is added to the discrete-time sliding mode, which prevents the saturation phenomenon. In [25], authors use the hyperbolic tangent function to process the system input and saturated input, and bring the error into the system dynamics equation to ensure the boundedness of the system input. In [26], an adaptive law is presented to reduce the adverse effect arising from input saturation based on a multiple operation solution, and the adaptive law is capable of converging to the specified ratio of the desired input to the saturation boundary while the closed-loop system stabilizes. However, from the existing research, there is no controller based on the NFTSM algorithm that considers the issue of input saturation. The reason may be that considering the saturation problem will greatly increase the difficulty of system stability analysis driven by the NFTSM controller. As far as we know, the design of the NFTSM controller being affected by saturated input is still an unsolved problem.

In the above articles, the form of the sliding mode surface is changed by using the saturation function when dealing with the input saturation problem. Inspired by them, in this paper, the SNFTSM control problem for the CFP system subject to input saturation is considered. In order to design the saturated controller, a new SNFTSM manifold composed of the NFTSM and the saturation function is proposed. Further, the saturation controller is brought into the system state equation, and the Lyapunov function is designed to verify the stability of the system. Different from NFTSM, due to the existence of saturation functions and signum functions, the SNFTSM controller stability analysis needs to be discussed in different situations. After discussion, it can be concluded that no matter where the initial state of the system is in the state’s phase plane, the system state can converge to the sliding mode surface in a finite time and move along the sliding mode surface to the equilibrium point. Finally, through simulation and comparison with NTSM, NFTSM and SNTSM algorithms, it is found that the SNFTSM algorithm can ensure the stable and efficient operation of the CFP system, and the amplitude of control input is much smaller than the other sliding mode algorithms.

## 2. Dynamic Modeling

**Figure 2:**
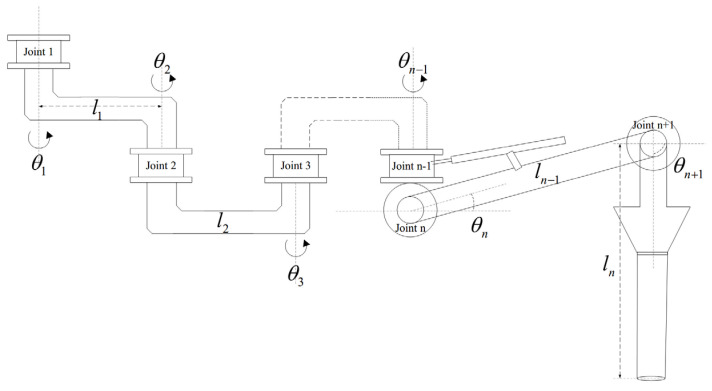
The n-link CFP model diagram.

The CFP system is connected by rigid pipelines, rigid joints and rigid elbows. As shown in Figure 1, the CFP model is relatively simple, consisting of two rigid pipelines and three rotary joints. In which the second rigid pipeline can rotate both horizontally and vertically. In this section, in order to demonstrate the universality of the trajectory tracking control algorithm in the CFP system, we establish a dynamic model of the n-link CFP system, as shown in Figure 2.

**Assumption 1.**  *Assuming that the density of all rigid pipelines and elbows in the CFP system is uniform, and the joint mass cannot be ignored but can be regarded as a particle.*

From Figure 2, we know that the CFP system consists of n rigid pipelines and *n* + 1 rotary joints. The first *n* − 1 rotary joints drive the first *n* − 1 rigid pipelines to rotate horizontally, and the nth joint drives the nth rigid pipeline to rotate vertically. At the same time, it can be seen from Figure 1 that the last joint has no driving device, but under the action of the support rod, the end pipeline is always kept perpendicular to the horizontal plane, that is $\theta_{n+1}\equiv \pi /4$. Therefore, combined with Assumption 1, it can be determined that the mass distribution of each section of pipeline is uniform and the center of mass is located in the middle of the pipeline. Further use the Euler–Lagrangian modeling method [25] to establish the dynamic model of the CFP system.

Define $m_{pi}$ is the mass of each pipeline, $m_{qj}$ is the mass of each joint, $l_{i}$ is the length of each pipeline and $r_{i}=1/2l_{i}$, where i=1,…,n,j=1,…,n+1. According to the Euler–Lagrangian modeling method, the total kinetic energy of the CFP system can first be obtained as:

(1) $$E_{k}=E_{kp}+E_{kq},$$

where $E_{kp}$ represents the total kinetic energy of all pipelines of CFP, and $E_{kq}$ denotes the total kinetic energy of all joints.

**Figure 3:**
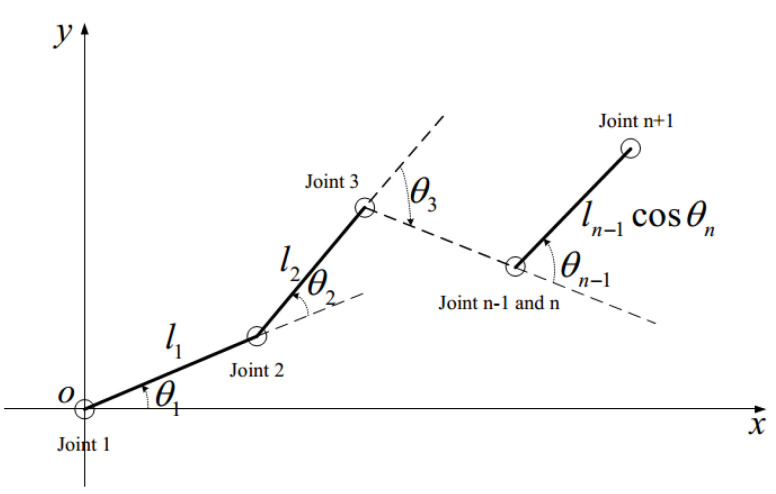
The n-link CFP planar figure.

Define $\left(x_{qj},y_{qj},z_{qj}\right)$ as the space coordinate of the jth joint mass center and $\left(x_{pi},y_{pi},z_{pi}\right)$ as the space coordinate of the ith pipeline mass center. From Figure 3, we can obtain the space coordinate of the joint mass center $\left(x_{qj},y_{qj},z_{qj}\right)$ as:

(2) $$\begin{array}{l} x_{qj}=\sum_{j=1}^{j}\left(l_{j-1}\cos(\theta_{1}+\cdot \cdot \cdot +\theta_{j-1})\right),\\ y_{qj}=\sum_{j=1}^{j}\left(l_{j-1}\sin(\theta_{1}+\cdot \cdot \cdot +\theta_{j-1})\right),\\ z_{qj}=z_{qj},\\ j=1,\ldots ,n-1,\\ x_{q(n+1)}=x_{qn}+l_{n-1}\cos\theta_{n}\cos(\theta_{1}+\cdot \cdot \cdot +\theta_{n-1}),\\ y_{q(n+1)}=y_{qn}+l_{n-1}\cos\theta_{n}\sin(\theta_{1}+\cdot \cdot \cdot +\theta_{n-1}),\\ z_{q(n+1)}=z_{qn}+r_{n-1}\sin\theta_{n}, \end{array}$$

where $z_{qj}$ is a constant. Since the nth joint and the (*n* − 1)th joint coincide in the XY plane, $\left(x_{qn},y_{qn})=(x_{q(n-1)},y_{q(n-1)}\right)$.

Because $\theta_{n+1}\equiv \pi /4$, we can get the last pipeline center of mass $\left(x_{pn},y_{pn},z_{pn})=(x_{qn},y_{qn},z_{qn}-r_{n}\right)$. The space coordinate of the other pipeline mass center $\left(x_{pi},y_{pi},z_{pi}\right)$ can be expressed as:

(3) $$\begin{array}{l} x_{pi}=x_{qi}+r_{i}\cos(\theta_{1}+\cdot \cdot \cdot +\theta_{i}),\\ y_{pi}=y_{qi}+r_{i}\sin(\theta_{1}+\cdot \cdot \cdot +\theta_{i}),\\ z_{pi}=z_{pi},i=1,\ldots ,n-2,\\ x_{p(n-1)}=x_{qn}+r_{n-1}\cos\theta_{n}\cos(\theta_{1}+\cdot \cdot \cdot +\theta_{n-1}),\\ y_{p(n-1)}=y_{qn}+r_{n-1}\cos\theta_{n}\sin(\theta_{1}+\cdot \cdot \cdot +\theta_{n-1}),\\ z_{p(n-1)}=z_{qn}+r_{n-1}\sin\theta_{n}. \end{array}$$

According to Formula (2) and Formula (3), we can get the velocity of each pipeline and joint as follows:

(4) $$\begin{array}{l} v_{pi}^{2}={\dot{x}}_{pi}^{2}+{\dot{y}}_{pi}^{2}+{\dot{z}}_{pi}^{2},\;\;\;\;\;i=1,\ldots ,n,\\ v_{qj}^{2}={\dot{x}}_{qj}^{2}+{\dot{y}}_{qj}^{2}+{\dot{z}}_{qj}^{2},\;\;\;\;\;j=1,\ldots ,n+1. \end{array}$$

Then, the kinetic energy of all pipelines and all joints can be expressed, respectively, as:

(5) $$\begin{array}{l} E_{kp}=\sum_{i=1}^{n}E_{kpi}=\sum_{i=1}^{n}\frac{1}{2}m_{pi}v_{pi}^{2},\\ E_{kq}=\sum_{j=1}^{n+1}E_{kqj}=\sum_{j=1}^{n+1}\frac{1}{2}m_{qj}v_{qj}^{2}. \end{array}$$

In order to establish the CFP dynamic model, it is also necessary to calculate the gravitational potential energy of the system. Since the gravitational potential energy of the first *n* − 2 pipelines and the first n joints is unchanged when the CFP system moves, the gravitational potential energy of the system can be expressed as:

(6) $$E_{g}=E_{gp(n-1)}+E_{gpn}+E_{gq(n+1)}=m_{p(n-1)}gr_{n-1}s_{n}+\left(m_{pn}+m_{q(n+1)}\right)gl_{n-1}s_{n},$$

where $s_{n}=1+sin\theta_{n}$.

According to the structure description of the CFP, it is known that the last joint has no driving device, so the driving torque of each CFP system joint the can be obtained according to the Lagrange formula:

(7) $$\tau_{i}=\frac{d}{dt}\left(\frac{\partial L}{\partial \theta_{i}}\right)+\frac{\partial L}{\partial \theta_{i}},\;\;\;\;i=1,\ldots ,n,$$

where $\tau ={ [\begin{array}{cccc} \tau_{1} & \tau_{2} & \cdot \cdot \cdot & \tau_{n} \end{array}]}^{T}$, $L=E_{k}-E_{p}$.

On the basis of Formulas (1)–(7), the dynamic model of the CFP system can be obtained as follows [27]:

(8) $$M(\theta )\ddot{\theta }+C(\theta ,\dot{\theta })\dot{\theta }+G(\theta )+f=\tau ,$$

where $\theta \in R^{n}$, $\dot{\theta }\in R^{n}$ and $\ddot{\theta }\in R^{n}$ represent the rotation angle, angular velocity and angular acceleration of each joint, respectively, $M(\theta )\in R^{n\times n}$ denotes the inertia matrix, $C(\theta ,\dot{\theta })\in R^{n\times n}$ represents the Coriolis force and centripetal force matrix, $G(\theta )\in R^{n}$ is the gravitational force, $f\in R^{n}$ denotes the friction and $\tau \in R^{n}$ represents the vector of control input.

The dynamic model of the CFP system described by Formula (8) has the following properties [28], which are useful to the subsequent controller design in this article:

**Property 1.**  *If*θ*and*$\dot{\theta }$*are uniformly bounded and continuous,*M(θ), $C(\theta ,\dot{\theta })$*and*G(θ)*are uniformly bounded and continuous.*

**Property 2.**  *The matrix* M(θ) *is positive definite, symmetric and bounded. Thus, there is a vector* $x\in R^{n}$ *such that the following inequality holds:*

$$0<\lambda_{\min}{\left\|x\right\|}^{2}\leq x^{T}M(\theta )x\leq \lambda_{\max}{\left\|x\right\|}^{2}<+\infty ,\;$$

*where*$\lambda_{\min}(\lambda_{\max})$*is the minimum (maximum) eigenvalue of*M(θ).

**Property 3.**  *The matrix is skew-symmetric, that is:*

$$y^{T} [\dot{M}(\theta )-2C(\theta ,\dot{\theta })]y=0,\;\forall y\in R^{n}.$$

## 3. Control Design and Stability Analysis

In this section, the content is divided into three parts. In the first part, we design the sliding mode surface of the SNFTSM algorithm, and analyze the advantages of the sliding mode surface proposed in this paper compared with the other terminal sliding mode surfaces. In the second part, according to the CFP system dynamic Equation (8), the trajectory tracking controller is designed using SNFTSM algorithm. In the last part, the stability of the system is analyzed using Lyapunov’s second method.

### 3.1. SNFTSM Algorithm

In this paper, we propose a SNFTSM algorithm to solve the trajectory tracking control problem for the CFP systems. The new sliding mode manifold is constructed by the NFTSM manifold, saturation functions and signum functions. The specific form of SNFTSM manifold is as follows:

(9) $$s=sat_{\bar{x}}(x)+\alpha sat_{\bar{x}}^{g/h}(x)+\beta sym^{p/q}(\dot{x}),$$

with

(10) $$sat_{\bar{x}}(x)=\left\{\begin{array}{l} {\bar{x}}^{2}\mathrm{sgn}(x),\;\;\;\;\;\;\;\;\;\;\;|x|\geq \bar{x},\\ -x^{2}\mathrm{sgn}(x)+2\bar{x}x,\;\;\;\;\;|\dot{x}|<\bar{x}, \end{array}\right.$$

(11) $$sym^{p/q}(\dot{x})=|\dot{x}{|}^{p/q}\mathrm{sgn}(\dot{x}),$$

where x denotes a state variable, α>0, β>0, p, q, g and h are all positive odd numbers and 1<p/q<2, g/h>p/q and sgn(⋅) expresses the signum function.

According to the expression of the SNFTSM manifold (9), it is obvious that it contains the form of the NFTSM manifold, so it also inherits the advantages of the NFTSM algorithm. Compared with the NTSM algorithm, the NFTSM has faster convergence rate. When the system state is close to the sliding surface, the higher order term of x is ignored. Thus, the NFTSM surface is similar to the NTSM surface. On the contrary, the higher-order term of x plays a leading role when the system state is far from the sliding surface and the approaching speed is greater than NTSM. At the same time, the form of NFTSM also effectively avoids the singularity of the controller. However, the shortcoming of the NFTSM algorithm is also obvious. Owing to the sliding mode s is related to the state error of the system, when the initial state of the system is far from the expected state, the controller output can easily exceed the upper limit of the actuator load.

The SNFTSM algorithm proposed in this paper can solve this problem well. Because of the existence of the saturation function, the system state error has a boundary value, so the sliding surface s has an upper bound, which can further effectively reduce the amplitude of the controller output and reduce the load intensity of the actuator.

### 3.2. Controller Design

For the dynamic model (8) of the CFP system, the system state error is defined as follows:

(12) $$e=\theta -\theta_{d},$$

where $\theta_{d}\in R^{n}$ represents the desired trajectory of each joint, and $e={ [\begin{array}{cccc} e_{1} & e_{2} & \cdot \cdot \cdot & e_{n} \end{array}]}^{T}\in R^{n}$ represents the trajectory tracking error.

According to Formulas (9) and (12), the SNFTSM manifold is designed as:

(13) $$s=sat_{\bar{e}}(e)+\alpha sat_{\bar{e}}^{g/h}(e)+\beta sym^{p/q}(\dot{e}),$$

where $s={\left[\begin{array}{ccc} s_{1} & \cdot \cdot \cdot & s_{n} \end{array}\right]}^{T}$, $\alpha =diag(\alpha_{i})$, $\beta =diag(\beta_{i})$, $\alpha_{i}>0$, $\beta_{i}>0$, i=1,2,…,n, $sat_{\bar{e}}(e)={\left[\begin{array}{ccc} sat_{{\bar{e}}_{1}}(e_{1}) & \cdots & sat_{{\bar{e}}_{n}}(e_{n}) \end{array}\right]}^{T}$, and satisfies:

(14) $$sat_{{\bar{e}}_{i}}(e_{i})=\left\{\begin{array}{l} {\bar{e}}_{i}^{2}\mathrm{sgn}(e_{i}),\;\;\;\;\;\;\;\;\;\;|e_{i}|\geq {\bar{e}}_{i}\\ -e_{i}^{2}\mathrm{sgn}(e_{i})+2{\bar{e}}_{i}e_{i},\;\;|e_{i}|<{\bar{e}}_{i} \end{array}\right..$$

For the CFP system, the upper bound of the position error is designed as ${\bar{e}}_{i}=1rad$. Meanwhile, $sym(\dot{e})={\left[\begin{array}{ccc} sym({\dot{e}}_{1}) & \cdots & sym({\dot{e}}_{n}) \end{array}\right]}^{T}$, and satisfies:

(15) $$sym^{p/q}({\dot{e}}_{i})=|{\dot{e}}_{i}{|}^{p/q}\mathrm{sgn}({\dot{e}}_{i}).$$

Since the SNFTSM algorithm is proposed for the first time, it is necessary to verify the convergence of the system state on the sliding mode surface. In this case, $s_{i}=0$, and we can obtain:

(16) $$sat_{{\bar{e}}_{i}}(e_{i})+\alpha_{i}sat_{{\bar{e}}_{i}}^{g/h}(e_{i})+\beta_{i}sym^{p/q}({\dot{e}}_{i})=0.$$

Bring Formulas (14) and (15) into above Formula:

(17) $${\dot{e}}_{i}=\left\{\begin{array}{l} -{\left(\frac{{\bar{e}}_{i}^{2}+\alpha_{i}{\bar{e}}_{i}^{2g/h}}{\beta_{i}}\right)}^{q/p}\mathrm{sgn}(e_{i}),\;\;\;\;\;\;\;\;|e_{i}|\geq {\bar{e}}_{i}\\ -{\left(\frac{\xi_{i}+\alpha_{i}\xi_{i}^{g/h}}{\beta_{i}}\right)}^{q/p}\mathrm{sgn}(e_{i}),\;\;\;\;\;\;\;\;\;|e_{i}|<{\bar{e}}_{i} \end{array}\right.,$$

where $\xi_{i}=2{\bar{e}}_{i}|e_{i}|-e_{i}^{2}$.

In order to prove that the system state can converge to the equilibrium point in finite time on the sliding mode surface, the finite time convergence lemma is introduced first.

**Lemma 1.**  *Assume that the density of all rigid pipelines and elbows in the CFP system is uniform, and the joint mass cannot be ignored but can be regarded as a particle. If the positive definite function V(t) satisfies the below inequality* [29]*:*

$$\dot{V}(t)\leq -\lambda V^{\gamma }(t),\;\forall t>t_{0},\;V(t_{0})>0,\;$$

*where* λ>0*,* 0<γ<1*, then there exists a* $t_{1}$ *guarantee that when* $t>t_{1}$*,* V(t)=0*, where the expression of* $t_{1}$ *is:*

$$t_{1}=t_{0}+\frac{V^{1-\gamma }(t_{0})}{\lambda (1-\gamma )}.\;$$

The Lyapunov function is considered as follows:

(18) $$V_{1}=\sum_{i=1}^{n}{\bar{V}}_{i},$$

where ${\bar{V}}_{i}=\frac{1}{2}e_{i}^{2}$.

Due to the existence of saturation functions, it is discussed in two cases. The first case is when $|e_{i}|\geq {\bar{e}}_{i}$:

(19) $$\dot{{\bar{V}}_{i}}=e_{i}{\dot{e}}_{i}=-{\left(\frac{{\bar{e}}_{i}^{2}+\alpha_{i}{\bar{e}}_{i}^{2g/h}}{\beta_{i}}\right)}^{q/p}|e_{i}|=-{\tilde{\delta }}_{i}V_{i}^{1/2},$$

where ${\tilde{\delta }}_{i}=\sqrt{2}{\left(\frac{{\bar{e}}_{i}^{2}+\alpha_{i}{\bar{e}}_{i}^{2g/h}}{\beta_{i}}\right)}^{q/p}$, owing to $\alpha_{i}>0$, $\beta_{i}>0$ and then ${\tilde{\delta }}_{i}>0$.

The second case is when $|e_{i}|<{\bar{e}}_{i}$:

(20) $$\dot{{\bar{V}}_{i}}=e_{i}{\dot{e}}_{i}=-{\left(\frac{\xi_{i}+\alpha_{i}\xi_{i}^{g/h}}{\beta_{i}}\right)}^{q/p}|e_{i}|=-{\bar{\delta }}_{i}V_{i}^{1/2},$$

where ${\bar{\delta }}_{i}=\sqrt{2}{\left(\frac{\xi_{i}+\alpha_{i}\xi_{i}^{g/h}}{\beta_{i}}\right)}^{q/p}$, because of $\xi_{i}=2{\bar{e}}_{i}|e_{i}|-e_{i}^{2}$ and $|e_{i}|<{\bar{e}}_{i}$, we can obtain ${\bar{\delta }}_{i}>0$ except $e_{i}=0$.

Define $\delta_{\min}=\min\{{\tilde{\delta }}_{1},\ldots ,{\tilde{\delta }}_{n},{\bar{\delta }}_{1},\ldots ,{\bar{\delta }}_{n}\}$, and the derivative of the Lyapunov function (18) can be obtained:

(21) $${\dot{V}}_{1}=\sum_{i=1}^{n}{\dot{\bar{V}}}_{i}\leq -\delta_{\min}\sum_{i=1}^{n}{\bar{V}}_{i}^{1/2}\leq -\delta_{\min}V_{1}^{1/2}.$$

According to Lemma 1, when the system state error reaches the sliding mode surface, it will converge to zero in finite time, and the convergence time is:

(22) $$t_{1}=t_{0}+\frac{2V_{1}^{1/2}(t_{0})}{\delta_{\min}}.$$

**Figure 4:**
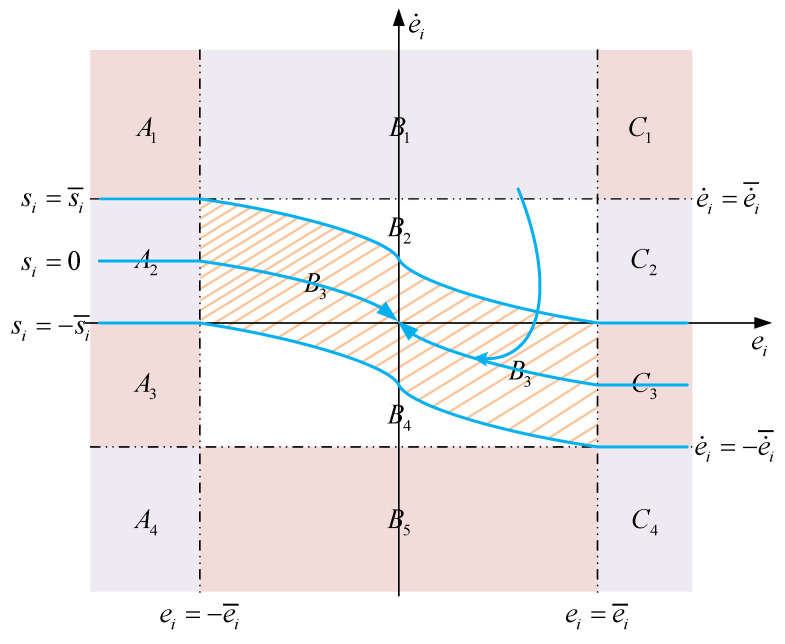
The phase plot of the SNFTSM manifold.

From the previous proof, it is known that when the system state error is on the sliding mode surface, it can converge to the coordinate origin in finite time. It is further necessary to discuss the convergence of the state error when the state error is not on the sliding mode surface, that is, s≠0. In order to ensure that the system state error can converge to the sliding mode surface, the controller based on the SNFTSM algorithm needs to be designed. Therefore, the upper bound of saturation function ${\bar{s}}_{i}$ needs to be designed. As shown in Figure 4, when $s_{i}={\bar{s}}_{i}$, $|e_{i}|>{\bar{e}}_{i}$, according to Formula (14), we can obtain $sat(e_{i})={\bar{e}}_{i}^{2}\mathrm{sgn}(e_{i})$. Then, design ${\bar{s}}_{i}$ as follows:

(23) $${\bar{s}}_{i}={\bar{e}}_{i}^{2}+\alpha {\bar{e}}_{i}^{2g/h}.$$

As shown in Figure 4, when $e_{i}=-{\bar{e}}_{i}$, $s_{i}={\bar{s}}_{i}$, it can be obtained from Formula (13) and Formula (23):

(24) $$s_{i}=-{\bar{e}}_{i}^{2}-\alpha_{i}{\bar{e}}_{i}^{2g/h}+\beta_{i}sym^{p/q}({\dot{e}}_{i})={\bar{e}}_{i}^{2}+\alpha {\bar{e}}_{i}^{2g/h}={\bar{s}}_{i}.$$

Further, we can get the expression of $\bar{{\dot{e}}_{i}}$:

(25) $${\dot{e}}_{i}={\left(\frac{2{\bar{e}}_{1}^{2}+2\alpha_{i}{\bar{e}}_{i}^{2g/h}}{\beta_{i}}\right)}^{q/p}={\bar{\dot{e}}}_{i}.$$

In the same form as the saturation function (14), $sat_{{\bar{\dot{e}}}_{i}}({\dot{e}}_{i})$ can be expressed as:

(26) $$sat_{{\bar{\dot{e}}}_{i}}({\dot{e}}_{i})=\left\{\begin{array}{l} {\bar{\dot{e}}}_{i}^{2}\mathrm{sgn}({\dot{e}}_{i}),\;\;\;\;\;\;\;\;\;\left|{\dot{e}}_{i}\right|>{\bar{\dot{e}}}_{i}\\ -{\dot{e}}_{i}^{2}\mathrm{sgn}({\dot{e}}_{i})+2{\bar{\dot{e}}}_{i}{\dot{e}}_{i},\;\left|{\dot{e}}_{i}\right|\leq {\bar{\dot{e}}}_{i} \end{array}\right..$$

Then, the controller is designed as follows:

(27) $$\begin{array}{ll} \tau \;= & -\frac{g}{h}\alpha M\zeta {\left|mtr(sat_{\bar{\dot{e}}}(\dot{e}))\right|}^{1-p/2q}mtr(sat_{\bar{e}}^{{}^{g/h-1}}(e))\mathrm{sgn}(s)\\ & -M\zeta {\left|mtr(sat_{\bar{\dot{e}}}(\dot{e}))\right|}^{1-p/2q}\mathrm{sgn}(s)-ksat_{{\bar{e}}^{2}+\alpha {\bar{e}}^{2g/h}}^{{}^{\frac{2q-p}{p}}}(s)\\ & +M{\ddot{\theta }}_{d}+C(\theta ,\dot{\theta })\dot{\theta }+G(\theta )+f, \end{array}$$

where $mtr(sat_{\bar{\dot{e}}}(\dot{e}))$ represents the diagonal matrix composed of the elements in vector $sat_{\bar{\dot{e}}}(\dot{e})$ in order, $\zeta =\frac{2q}{p}\beta^{-1}$.

Since α, ζ and mtr(⋅) are all diagonal matrices, according to Formula (27), the expression of $\tau_{i}$ can be written as:

(28) $$\begin{array}{ll} \tau_{i} & =-\frac{g}{h}\alpha_{i}M_{ii}\zeta_{i}{\left|sat_{{\bar{\dot{e}}}_{i}}({\dot{e}}_{i})\right|}^{1-p/2q}sat_{{\bar{e}}_{i}}^{{}^{g/h-1}}\mathrm{sgn}(s_{i})-M_{ii}\zeta_{i}{\left|sat_{{\bar{\dot{e}}}_{i}}({\dot{e}}_{i})\right|}^{1-p/2q}\mathrm{sgn}(s_{i})\\ & \;\;\;\;-ksat_{{\bar{e}}_{i}^{2}+\alpha_{i}{\bar{e}}_{i}^{2g/h}}^{{}^{\frac{2q-p}{p}}}(s_{i})+M_{i,}{\ddot{\theta }}_{d}+C_{i,}(\theta ,\dot{\theta })\dot{\theta }+G_{i}(\theta )+f_{i}\\ & ⩽M_{ii}\zeta_{i}{\left(\frac{2{\bar{e}}_{i}^{2}+2\alpha_{i}{\bar{e}}_{i}^{2g/h}}{\beta_{i}}\right)}^{\frac{2q-p}{p}}\left(1+\frac{\alpha_{i}g}{h}{\bar{e}}_{i}^{2g/h-2}\right)+k{\left({\bar{e}}_{i}^{2}+\alpha_{i}{\bar{e}}_{i}^{2g/h}\right)}^{\frac{2q-p}{p}}\\ & \;\;\;\;+M_{i,}{\ddot{\theta }}_{d}+C_{i,}(\theta ,\dot{\theta })\dot{\theta }+G_{i}(\theta )+f_{i}\\ & ⩽\tau_{i\max}, \end{array}$$

where $M_{i,\cdot }$ and $C_{i,\cdot }$ represent the row i of matrices M and C, respectively. $M_{ii}$ represents the diagonal element in line i.

### 3.3. Stability Analysis

By dynamics model (8) and controller (27), we can get:

(29) $$\begin{array}{lll} \ddot{e} & = & \ddot{\theta }-{\ddot{\theta }}_{d}\\ & = & M^{-1}(\tau -C\dot{\theta }-G-f)-{\ddot{\theta }}_{d}\\ & = & -\frac{g}{h}\alpha \zeta {\left|mtr(sat_{\bar{\dot{e}}}(\dot{e}))\right|}^{1-p/2q}mtr(sat_{\bar{e}}^{g/h-1}(e))\mathrm{sgn}(s)\\ & & -\zeta {\left|mtr(sat_{\bar{\dot{e}}}(\dot{e}))\right|}^{1-p/2q}\mathrm{sgn}(s)-ksat_{\bar{s}}^{{}^{\frac{2q-p}{p}}}(s). \end{array}$$

Since all matrices except $M^{-1}$ in Formula (29) are diagonal matrices, the expression of ${\ddot{e}}_{i}$ can be written as:

(30) $$\begin{array}{l} {\ddot{e}}_{i}=-\frac{g\alpha_{i}}{h}\zeta_{i}{\left|sat_{{\bar{\dot{e}}}_{i}}({\dot{e}}_{i})\right|}^{1-p/2q}sat_{{\bar{e}}_{i}}^{g/h-1}(e_{i})\mathrm{sgn}(s_{i})\\ \;\;\;\;-\zeta_{i}{\left|sat_{{\bar{\dot{e}}}_{i}}({\dot{e}}_{i})\right|}^{1-p/2q}\mathrm{sgn}(s_{i})-ksat_{{\bar{s}}_{i}}^{{}^{\frac{2q-p}{p}}}(s_{i}). \end{array}$$

In order to verify whether the system state error can converge to the sliding mode surface, the Lyapunov function is selected as follows:

(31) $${\tilde{V}}_{i}=\frac{1}{2}s_{i}^{2},\;\;\;\;i=1,2,\ldots ,n.$$

Take the derivative of $s_{i}$ and bring Formula (30) into it to obtain:

(32) $$\begin{array}{lll} {\dot{s}}_{i} & = & s\dot{a}t_{{\bar{e}}_{i}}(e_{i})+\frac{\alpha_{i}g}{h}sat_{{\bar{e}}_{i}}^{g/h-1}(e_{i})s\dot{a}t_{{\bar{e}}_{i}}(e_{i})+\frac{\beta_{i}p}{q}{\dot{e}}_{i}^{p/q-1}{\ddot{e}}_{i}\\ & = & s\dot{a}t_{{\bar{e}}_{i}}(e_{i})+\frac{\alpha_{i}g}{h}sat_{{\bar{e}}_{i}}^{g/h-1}(e_{i})s\dot{a}t_{{\bar{e}}_{i}}(e_{i})-\frac{2\alpha_{i}g}{h}{\dot{e}}_{i}{}^{p/q-1}sat_{{\bar{e}}_{i}}^{g/h-1}(e_{i}){\left|sat_{{\bar{\dot{e}}}_{i}}({\dot{e}}_{i})\right|}^{1-p/2q}\mathrm{sgn}(s_{i})\\ & & -2{\dot{e}}_{i}{}^{p/q-1}{\left|sat_{{\bar{\dot{e}}}_{i}}({\dot{e}}_{i})\right|}^{1-p/2q}\mathrm{sgn}(s_{i})-\frac{\beta_{i}pk}{q}{\dot{e}}_{i}{}^{p/q-1}sat_{{\bar{s}}_{i}}^{{}^{\frac{2q-p}{p}}}(s_{i}). \end{array}$$

By deriving Formula (14), we can get the expression of $s\dot{a}t_{{\bar{e}}_{i}}(e_{i})$ as follows:

(33) $$s\dot{a}t_{{\bar{e}}_{i}}(e_{i})=\left\{\begin{array}{l} 0,\;\;\;\;\;\;\;\;\;\;\;|e_{i}|\geq {\bar{e}}_{i}\\ 2{\bar{e}}_{i}{\dot{e}}_{i}-2|e_{i}|{\dot{e}}_{i},\;\;\;|e_{i}|<{\bar{e}}_{i} \end{array}\right..$$

As shown in Figure 4, when $(e_{i},{\dot{e}}_{i})\subset A_{1}\cup A_{4}\cup C_{1}\cup C_{4}$, $|e_{i}|\geq {\bar{e}}_{i}$, $|{\dot{e}}_{i}|\geq \bar{{\dot{e}}_{i}}$ and $|s_{i}|\geq {\bar{s}}_{i}$, Formula (32) can be rewritten as:

(34) $${\dot{s}}_{i}=-{\dot{e}}_{i}{}^{p/q-1}{\bar{\dot{e}}}_{i}^{2-p/q}\left(2+\frac{2\alpha_{i}g}{h}{\bar{e}}_{i}^{2g/h-2}\right)\mathrm{sgn}(s_{i})-\frac{\beta_{i}pk}{q}{\dot{e}}_{i}{}^{p/q-1}{\bar{s}}_{i}^{\frac{4q-2p}{p}}\mathrm{sgn}(s_{i}).$$

Take the derivative of Formula (31) and bring Formula (34) into ${\dot{\tilde{V}}}_{i}$:

(35) $$\begin{array}{l} {\dot{\tilde{V}}}_{i}=-{\dot{e}}_{i}{}^{p/q-1}{\bar{\dot{e}}}_{i}^{2-p/q}\left(2+\frac{2\alpha_{i}g}{h}{\bar{e}}_{i}^{2g/h-2}\right)|s_{i}|-\frac{\beta_{i}pk}{q}{\dot{e}}_{i}{}^{p/q-1}{\bar{s}}_{i}^{\frac{4q-2p}{p}}|s_{i}|\\ \;\;\leq -\frac{\beta_{i}pk}{q}{\dot{e}}_{i}{}^{p/q-1}{\bar{s}}_{i}^{\frac{4q-2p}{p}}|s_{i}|\\ \;\;\leq -{\bar{\varepsilon }}_{i}{\tilde{V}}_{i}^{1/2}, \end{array}$$

where ${\bar{\varepsilon }}_{i}=\sqrt{2}\frac{\beta_{i}pk}{q}{\dot{e}}_{i}{}^{p/q-1}{\bar{s}}_{i}^{\frac{4q-2p}{p}}$, among them $\beta_{i}$, p, q and k are all positive numbers, and $|{\dot{e}}_{i}|\geq {\bar{\dot{e}}}_{i}>0$, thus ${\bar{\varepsilon }}_{i}>0$. According to Lemma 1, when $(e_{i},{\dot{e}}_{i})\subset A_{1}\cup A_{4}\cup C_{1}\cup C_{4}$, the system state error will converge to the sliding mode surface in a finite time.

Then, when $(e_{i},{\dot{e}}_{i})\subset A_{2}\cup C_{3}$, $|e_{i}|\geq {\bar{e}}_{i}$, $0<|{\dot{e}}_{i}|<{\bar{\dot{e}}}_{i}$ and $0<|s_{i}|<{\bar{s}}_{i}$, Formula (27) can be rewritten as:

(36) $$\begin{array}{lll} {\dot{s}}_{i} & = & -2{\dot{e}}_{i}{}^{p/q-1}\varsigma_{i}\mathrm{sgn}(s_{i})-\frac{2\alpha_{i}g}{h}{\dot{e}}_{i}{}^{p/q-1}\varsigma_{i}{\bar{e}}_{i}^{2g/h-2}\mathrm{sgn}(s_{i})\\ & & -\frac{\beta_{i}pk}{q}{\dot{e}}_{i}{}^{p/q-1}{\left(-s_{i}^{2}\mathrm{sgn}(s_{i})+2{\bar{s}}_{i}s_{i}\right)}^{\frac{2q-p}{p}}, \end{array}$$

where $\varsigma_{i}={\left|-{\dot{e}}_{i}^{2}\mathrm{sgn}({\dot{e}}_{i})+2{\bar{\dot{e}}}_{i}{\dot{e}}_{i}\right|}^{1-p/2q}>0$.

Bring Formula (36) into ${\dot{\tilde{V}}}_{i}$:

(37) $$\begin{array}{lll} {\dot{\tilde{V}}}_{i} & = & -2{\dot{e}}_{i}{}^{p/q-1}\varsigma_{i}|s_{i}|-\frac{2\alpha_{i}g}{h}{\dot{e}}_{i}{}^{p/q-1}\varsigma_{i}{\bar{e}}_{i}^{2g/h-2}|s_{i}|-\frac{\beta_{i}pk}{q}{\dot{e}}_{i}{}^{p/q-1}{\left(-s_{i}^{2}+2{\bar{s}}_{i}|s_{i}|\right)}^{\frac{2q-p}{p}}|s_{i}|\\ & \leq & -\frac{\beta_{i}pk}{q}{\dot{e}}_{i}{}^{p/q-1}{\left(-s_{i}^{2}+2{\bar{s}}_{i}|s_{i}|\right)}^{\frac{2q-p}{p}}|s_{i}|\\ & = & -{\tilde{\varepsilon }}_{i}{\tilde{V}}_{i}^{1/2}, \end{array}$$

where ${\tilde{\varepsilon }}_{i}=\sqrt{2}\frac{\beta_{i}pk}{q}{\dot{e}}_{i}{}^{p/q-1}{\left(-s_{i}^{2}+2{\bar{s}}_{i}|s_{i}|\right)}^{\frac{2q-p}{p}}$. It is known that $|{\dot{e}}_{i}|>0$, $0<|s_{i}|<{\bar{s}}_{i}$, so ${\tilde{\varepsilon }}_{i}>0$. According to Lemma 1, when $(e_{i},{\dot{e}}_{i})\subset A_{2}\cup C_{3}$, the system state error will converge to the sliding mode surface in a finite time.

Further, when $(e_{i},{\dot{e}}_{i})\subset A_{3}\cup C_{2}$, $|e_{i}|\geq {\bar{e}}_{i}$, $0<|{\dot{e}}_{i}|<{\bar{\dot{e}}}_{i}$ and $|s_{i}|>{\bar{s}}_{i}$, the expression of ${\dot{s}}_{i}$ can be obtained:

(38) $${\dot{s}}_{i}=-2{\dot{e}}_{i}{}^{p/q-1}\varsigma_{i}\mathrm{sgn}(s_{i})-\frac{2\alpha_{i}g}{h}{\dot{e}}_{i}{}^{p/q-1}\varsigma_{i}{\bar{e}}_{i}^{2g/h-2}\mathrm{sgn}(s_{i})\;-\frac{\beta_{i}pk}{q}{\dot{e}}_{i}{}^{p/q-1}{\bar{s}}_{i}^{\frac{4q-2p}{p}}\mathrm{sgn}(s_{i}).$$

Bring Formula (38) into ${\dot{\tilde{V}}}_{i}$:

(39) $$\begin{array}{lll} {\dot{\tilde{V}}}_{i} & = & -2{\dot{e}}_{i}{}^{p/q-1}\varsigma_{i}|s_{i}|-\frac{2\alpha_{i}g}{h}{\dot{e}}_{i}{}^{p/q-1}\varsigma_{i}{\bar{e}}_{i}^{2g/h-2}|s_{i}|-\frac{\beta_{i}pk}{q}{\dot{e}}_{i}{}^{p/q-1}{\bar{s}}_{i}^{\frac{4q-2p}{p}}|s_{i}|\\ & \leq & -\frac{\beta_{i}pk}{q}{\dot{e}}_{i}{}^{p/q-1}{\bar{s}}_{i}^{\frac{4q-2p}{p}}|s_{i}|\\ & = & -{\bar{\varepsilon }}_{i}{\tilde{V}}_{i}^{1/2} \end{array}$$

It can be known from Formula (35) that ${\bar{\varepsilon }}_{i}>0$. And then, According to Lemma 1, when $(e_{i},{\dot{e}}_{i})\subset A_{3}\cup C_{2}$, the system state error will converge to the sliding mode surface in a finite time.

From the previous derivation process, it can be concluded that when $(e_{i},{\dot{e}}_{i})\subset A_{1-4}\cup C_{1-4}$, the system state error can converge to the sliding mode surface in a finite time, but this conclusion cannot be reached when $|e_{i}|\geq {\bar{e}}_{i}$ and ${\dot{e}}_{i}=0$. Therefore, it is further discussed whether the system state error can converge to the sliding mode surface in this case. When $\dot{e}=0$, Formula (30) can be expressed as:

(40) $$\begin{array}{l} {\ddot{e}}_{i}=-\frac{g\alpha_{i}}{h}\zeta_{i}{\left|sat_{{\bar{\dot{e}}}_{i}}({\dot{e}}_{i})\right|}^{1-p/2q}sat_{{\bar{e}}_{i}}^{g/h-1}(e_{i})\mathrm{sgn}(s_{i})\\ \;\;\;-\zeta_{i}{\left|sat_{{\bar{\dot{e}}}_{i}}({\dot{e}}_{i})\right|}^{1-p/2q}\mathrm{sgn}(s_{i})-ksat_{{\bar{s}}_{i}}^{(2q-p)/p}(s_{i})\\ \;=-ksat_{{\bar{s}}_{i}}^{(2q-p)/p}(s_{i})\\ \;=-k{\bar{s}}_{i}^{(4q-2p)/p}{\left(\mathrm{sgn}(s_{i})\right)}^{(2q-p)/p} \end{array}$$

It can be seen in Formula (40) and Figure 4, when $e_{i}>{\bar{e}}_{i}$ and $s_{i}={\bar{s}}_{i}$, ${\ddot{e}}_{i}<0$, the system state error will move down to the $C_{3}$ area; when $e_{i}<-{\bar{e}}_{i}$ and $s_{i}=-{\bar{s}}_{i}$, ${\ddot{e}}_{i}>0$, the system state error will move down to the $A_{2}$ area. Therefore, when $|e_{i}|\geq {\bar{e}}_{i}$, the system state error must converge to the sliding mode surface in a finite time.

Next, we need to discuss the convergence of system state error when $|e_{i}|<{\bar{e}}_{i}$. First, when $(e_{i},{\dot{e}}_{i})\subset B_{3}$, $|e_{i}|<{\bar{e}}_{i}$, $0<|{\dot{e}}_{i}|<{\bar{\dot{e}}}_{i}$ and $0<|s_{i}|<{\bar{s}}_{i}$, ${\dot{s}}_{i}$ can be rewritten as:

(41) $$\begin{array}{l} {\dot{s}}_{i}=\left(\frac{\alpha_{i}g}{h}sat_{{\bar{e}}_{i}}^{g/h-1}(e_{i})+1\right)\left(2{\bar{e}}_{i}{\dot{e}}_{i}-2|e_{i}|{\dot{e}}_{i}\right)\\ \;\;\;-{\dot{e}}_{i}{}^{p/q-1}\varsigma_{i}\left(2+\frac{2\alpha_{i}g}{h}sat_{{\bar{e}}_{i}}^{g/h-1}(e_{i})\right)\mathrm{sgn}(s_{i})\\ \;\;\;-\frac{\beta_{i}pk}{q}{\dot{e}}_{i}{}^{p/q-1}{\left(-s_{i}^{2}\mathrm{sgn}(s_{i})+2{\bar{s}}_{i}s_{i}\right)}^{\frac{2q-p}{p}}, \end{array}$$

where

$\varsigma_{i}={\left|-{\dot{e}}_{i}^{2}\mathrm{sgn}({\dot{e}}_{i})+2{\bar{\dot{e}}}_{i}{\dot{e}}_{i}\right|}^{1-p/2q}=|{\dot{e}}_{i}{|}^{2-p/q}{\left|-\mathrm{sgn}({\dot{e}}_{i})+2\frac{{\bar{\dot{e}}}_{i}}{{\dot{e}}_{i}}\right|}^{1-p/2q}=|{\dot{e}}_{i}{|}^{2-p/q}{\bar{\varsigma }}_{i}.$

Then, Formula (41) can be rewritten:

(42) $$\begin{array}{l} {\dot{s}}_{i}\;=\left(\frac{2\alpha_{i}g}{h}sat_{{\bar{e}}_{i}}^{g/h-1}(e_{i})+2\right)\left({\bar{e}}_{i}-|e_{i}|\right){\dot{e}}_{i}-|{\dot{e}}_{i}|{\bar{\varsigma }}_{i}\left(2+\frac{2\alpha_{i}g}{h}sat_{{\bar{e}}_{i}}^{g/h-1}(e_{i})\right)\mathrm{sgn}(s_{i})\\ \;\;\;-\frac{\beta_{i}pk}{q}{\dot{e}}_{i}{}^{p/q-1}{\left(-s_{i}^{2}+2{\bar{s}}_{i}|s_{i}|\right)}^{\frac{2q-p}{p}}\mathrm{sgn}(s_{i}). \end{array}$$

Bring Formula (42) into ${\dot{\tilde{V}}}_{i}$:

(43) $$\begin{array}{l} {\dot{\tilde{V}}}_{i}=\left(\frac{2\alpha_{i}g}{h}sat_{{\bar{e}}_{i}}^{g/h-1}(e_{i})+2\right)\left({\bar{e}}_{i}-|e_{i}|\right){\dot{e}}_{i}s_{i}-|{\dot{e}}_{i}|{\bar{\varsigma }}_{i}\left(2+\frac{2\alpha_{i}g}{h}sat_{{\bar{e}}_{i}}^{g/h-1}(e_{i})\right)|s_{i}|\\ \;\;\;\;-\frac{\beta_{i}pk}{q}{\dot{e}}_{i}{}^{p/q-1}{\left(-s_{i}^{2}+2{\bar{s}}_{i}|s_{i}|\right)}^{\frac{2q-p}{p}}|s_{i}|\\ \;\leq \left(\frac{2\alpha_{i}g}{h}sat_{{\bar{e}}_{i}}^{g/h-1}(e_{i})+2\right)\left({\bar{e}}_{i}-|e_{i}|\right)|{\dot{e}}_{i}||s_{i}|-|{\dot{e}}_{i}|{\bar{\varsigma }}_{i}\left(2+\frac{2\alpha_{i}g}{h}sat_{{\bar{e}}_{i}}^{g/h-1}(e_{i})\right)|s_{i}|\\ \;\;\;\;-\frac{\beta_{i}pk}{q}{\dot{e}}_{i}{}^{p/q-1}{\left(-s_{i}^{2}+2{\bar{s}}_{i}|s_{i}|\right)}^{\frac{2q-p}{p}}|s_{i}|\\ \;=\left(\frac{2\alpha_{i}g}{h}sat_{{\bar{e}}_{i}}^{g/h-1}(e_{i})+2\right)|{\dot{e}}_{i}||s_{i}|\rho_{i}-\frac{\beta_{i}pk}{q}{\dot{e}}_{i}{}^{p/q-1}{\left(-s_{i}^{2}+2{\bar{s}}_{i}|s_{i}|\right)}^{\frac{2q-p}{p}}|s_{i}|, \end{array}$$

where

$\rho_{i}=\left(\left({\bar{e}}_{i}-|e_{i}|\right)-{\bar{\varsigma }}_{i}\right),\;{\bar{\varsigma }}_{i}={\left|-\mathrm{sgn}({\dot{e}}_{i})+2\frac{{\bar{\dot{e}}}_{i}}{{\dot{e}}_{i}}\right|}^{1-p/2q},$owing to ${\bar{e}}_{i}=1$ and $0<|e_{i}|<{\bar{e}}_{i}$, it can be seen that ${\bar{e}}_{i}-|e_{i}|<1$. Further, when ${\dot{e}}_{i}>0$, ${\bar{\dot{e}}}_{i}/{\dot{e}}_{i}>1$, $\mathrm{sgn}({\dot{e}}_{i})=1$, and then ${\bar{\varsigma }}_{i}>1$; when ${\dot{e}}_{i}<0$, ${\bar{\dot{e}}}_{i}/{\dot{e}}_{i}<-1$, $\mathrm{sgn}({\dot{e}}_{i})=-1$, and then ${\bar{\varsigma }}_{i}>1$. It is known that 0<1−p/2q<1, so $\rho_{i}<0$, then we can obtain:

(44) $${\tilde{V}}_{i}\leq -\frac{\beta_{i}pk}{q}{\dot{e}}_{i}{}^{p/q-1}{\left(-s_{i}^{2}+2{\bar{s}}_{i}|s_{i}|\right)}^{\frac{2q-p}{p}}|s_{i}|=-{\tilde{\varepsilon }}_{i}{\tilde{V}}_{i}^{1/2}.$$

Because of $0<|{\dot{e}}_{i}|<{\bar{\dot{e}}}_{i}$ and $0<|s_{i}|<{\bar{s}}_{i}$, ${\tilde{\varepsilon }}_{i}>0$. Thus, the system state error will converge to the sliding mode surface in a finite time when $(e_{i},{\dot{e}}_{i})\subset B_{3}$ except ${\dot{e}}_{i}=0$.

Then, when $|e_{i}|<{\bar{e}}_{i}$, ${\dot{e}}_{i}=0$ and $|s_{i}|<{\bar{s}}_{i}$

(45) $${\ddot{e}}_{i}=-ksat_{{\bar{s}}_{i}}^{(2q-p)/p}(s_{i})=-k{\left(-s_{i}^{2}\mathrm{sgn}(s_{i})+2{\bar{s}}_{i}s_{i}\right)}^{\frac{2q-p}{p}},$$

when $s_{i}>0$, ${\ddot{e}}_{i}<0$ and $s_{i}<0$, ${\ddot{e}}_{i}>0$. Therefore, it can be seen from Figure 4 that when the system state error is in $B_{3}$, it can converge to the sliding mode surface.

Further, when $(e_{i},{\dot{e}}_{i})\subset B_{2}\cup B_{4}$, $|e_{i}|<{\bar{e}}_{i}$, $0<|{\dot{e}}_{i}|<{\bar{\dot{e}}}_{i}$ and $|s_{i}|\geq {\bar{s}}_{i}$, Formula (32) can be expressed as:

(46) $$\begin{array}{l} {\dot{s}}_{i}=\left(\frac{\alpha_{i}g}{h}sat_{{\bar{e}}_{i}}^{g/h-1}(e_{i})+1\right)\left(2{\bar{e}}_{i}{\dot{e}}_{i}-2|e_{i}|{\dot{e}}_{i}\right)-|e_{i}|{\bar{\varsigma }}_{i}\left(2+\frac{2\alpha_{i}g}{h}sat_{{\bar{e}}_{i}}^{g/h-1}(e_{i})\right)\mathrm{sgn}(s_{i})\\ \;\;\;\;\;\;-\frac{\beta_{i}pk}{q}|e_{i}{|}^{p/q-1}{\bar{s}}_{i}^{\frac{4q-2p}{p}}\mathrm{sgn}(s_{i}). \end{array}$$

Bring Formula (46) into ${\dot{\tilde{V}}}_{i}$:

(47) $$\begin{array}{l} {\dot{\tilde{V}}}_{i}=\left(\frac{2\alpha_{i}g}{h}sat_{{\bar{e}}_{i}}^{g/h-1}(e_{i})+2\right)\left({\bar{e}}_{i}-|e_{i}|\right){\dot{e}}_{i}s_{i}-|e_{i}|{\bar{\varsigma }}_{i}\left(2+\frac{2\alpha_{i}g}{h}sat_{{\bar{e}}_{i}}^{g/h-1}(e_{i})\right)\mathrm{sgn}(s_{i})s_{i}\\ \;\;\;\;-\frac{\beta_{i}pk}{q}e_{i}{}^{p/q-1}{\bar{s}}_{i}^{\frac{4q-2p}{p}}\mathrm{sgn}(s_{i})s_{i}\\ \;\leq \left(\frac{2\alpha_{i}g}{h}sat_{{\bar{e}}_{i}}^{g/h-1}(e_{i})+2\right)\left({\bar{e}}_{i}-|e_{i}|\right)|{\dot{e}}_{i}||s_{i}|-|e_{i}|{\bar{\varsigma }}_{i}\left(2+\frac{2\alpha_{i}g}{h}sat_{{\bar{e}}_{i}}^{g/h-1}(e_{i})\right)|s_{i}|\\ \;\;\;\;-\frac{\beta_{i}pk}{q}e_{i}{}^{p/q-1}{\bar{s}}_{i}^{\frac{4q-2p}{p}}|s_{i}|\\ \;\leq -\frac{\beta_{i}pk}{q}e_{i}{}^{p/q-1}{\bar{s}}_{i}^{\frac{4q-2p}{p}}|s_{i}|\\ \;=-\sqrt{2}\frac{\beta_{i}pk}{q}e_{i}{}^{p/q-1}{\bar{s}}_{i}^{\frac{4q-2p}{p}}V_{2}^{1/2}=-{\bar{\varepsilon }}_{i}{\tilde{V}}_{i}^{1/2}, \end{array}$$

where ${\bar{\varepsilon }}_{i}=\sqrt{2}\frac{\beta_{i}pk}{q}{\dot{e}}_{i}{}^{p/q-1}{\bar{s}}_{i}^{\frac{4q-2p}{p}}>0$. According to Lemma 1, when $(e_{i},{\dot{e}}_{i})\subset B_{2}\cup B_{4}$, the system state error will converge to the sliding mode surface in a finite time.

Finally, when $(e_{i},{\dot{e}}_{i})\subset B_{1}$, $|e_{i}|<{\bar{e}}_{i}$, $|{\dot{e}}_{i}|\geq {\bar{\dot{e}}}_{i}$ and $|s_{i}|\geq {\bar{s}}_{i}$, Formula (30) can be obtained:

(48) $${\ddot{e}}_{i}=-\frac{2gq\alpha_{i}}{hp\beta_{i}}{\left|sat_{{\bar{\dot{e}}}_{i}}({\dot{e}}_{i})\right|}^{1-p/2q}sat_{{\bar{e}}_{i}}^{g/h-1}(e_{i})\mathrm{sgn}(s_{i})-\frac{2q}{p\beta_{i}}{\left|sat_{{\bar{\dot{e}}}_{i}}({\dot{e}}_{i})\right|}^{1-p/2q}\mathrm{sgn}(s_{i})-ksat_{{\bar{s}}_{i}}^{\frac{2q-p}{p}}(s_{i}),$$

where p, q, g, h, $\alpha_{i}$ and $\beta_{i}$ are all positive numbers, g−h is an even number, so $sat_{{\bar{e}}_{i}}^{g/h-1}(e_{i})⩾0$. Additionally, because of $(e_{i},{\dot{e}}_{i})\subset B_{1}$, $sgn(s_{i})=1$, ${\bar{s}}_{i}={\bar{e}}_{i}{}^{2}+\alpha {\bar{e}}_{i}{}^{2g/h}$, and then Formula (48) can be expressed as the following inequality structure:

(49) $${\ddot{e}}_{i}⩽-ksat_{{\bar{s}}_{i}}^{\frac{2q-p}{p}}(s_{i})=-k{\left({\bar{e}}_{i}{}^{2}+\alpha {\bar{e}}_{i}{}^{2g/h}\right)}^{\frac{2q-p}{p}}.$$

Then, we can obtain:

(50) $$\dot{e}(t)\leq \dot{e}(0)-k{\left({\bar{e}}^{2}+\alpha {\bar{e}}^{2g/h}\right)}^{\frac{2q-p}{p}}t,$$

so it has T so that when t>T, $\dot{e}(t)<\bar{\dot{e}}$, and then the state error enters into $B_{2}$.

Similarly, when $(e_{i},{\dot{e}}_{i})\subset B_{5}$, there is T so that when t>T, $\dot{e}(t)>-\bar{\dot{e}}$, and then the state error enters into $B_{4}$.

At last, define $\varepsilon_{\min}=\min\{{\bar{\varepsilon }}_{1},\ldots ,{\bar{\varepsilon }}_{n},{\tilde{\varepsilon }}_{1},\ldots ,{\tilde{\varepsilon }}_{n}\}$, and then select the Lyapunov function:

(51) $$V_{2}=\sum_{i=1}^{n}{\tilde{V}}_{i}.$$

When $\left(e_{i},{\dot{e}}_{i})\in R^{2}\backslash (B_{1}\cup B_{5}\right)$, the derivative of $V_{2}$ can be obtained:

(52) $${\dot{V}}_{2}=\sum_{i=1}^{n}{\dot{\tilde{V}}}_{i}\leq -\varepsilon_{\min}\sum_{i=1}^{n}{\tilde{V}}_{i}^{1/2}\leq -\varepsilon_{\min}V_{2}^{1/2}.$$

According to Lemma 1, the CFP system states errors will converge to the sliding mode surface in a finite time when $\left(e_{i},{\dot{e}}_{i})\in R^{2}\backslash (B_{1}\cup B_{5}\right)$.

When $(e_{i},{\dot{e}}_{i})\in B_{1}\cup B_{5}$, according to Formulas (49) and (50), the CFP system states errors will approach the adjacent areas, and then the state error will arrive $\left(e_{i},{\dot{e}}_{i})\in R^{2}\backslash (B_{1}\cup B_{5}\right)$, and then converge to the sliding mode surface in finite time.

## 4. Simulation

**Figure 5:**
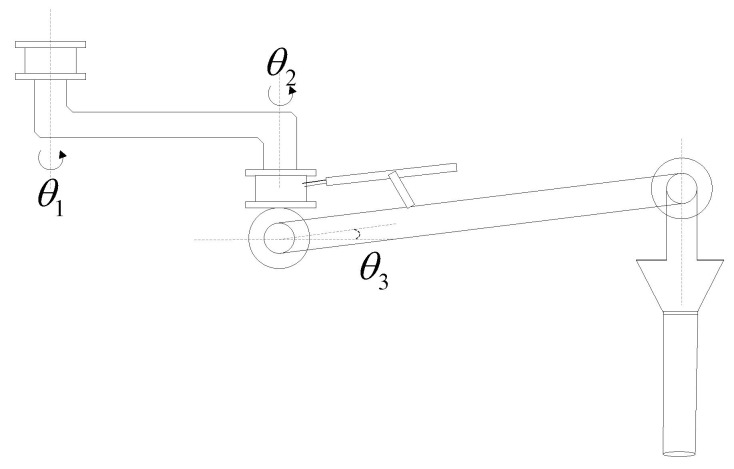
The 3-DOF CFP structure diagram.

In this section, we consider the 3-DOF CFP system shown in Figure 5 to simulate and verify the algorithm proposed in this paper. We define:

$$\theta ={\left[\begin{array}{ccc} \theta_{1} & \theta_{2} & \theta_{3} \end{array}\right]}^{T},$$

where $\theta_{1}$ and $\theta_{2}$ represent the horizontal rotation angles of the first two joints, and $\theta_{3}$ denotes the vertical rotation angle of the last joint.

The simulation parameters of the 3-DOF CFP are designed as follows:

$$l_{1}=1\;m,\;l_{2}=1\;m,\;m_{q1}=1\;\mathrm{kg},\;m_{q2}=1\;\mathrm{kg},\;m_{q3}=1\;\mathrm{kg},\;m_{p1}=10\;\mathrm{kg},\;m_{p2}=10\;\mathrm{kg},\;m_{p3}=1\;\mathrm{kg}.$$

For the daily operation of CFP automatic alignment refueling, it is fixed in expected position alignment, that is, after the tank car stops at the predetermined position, CFP carries out automatic alignment. Therefore, in order to verify the superiority of the algorithm, simulation verification is carried out for the fixed desired position. Design the fixed desired position and the initial position first:

$$\theta_{d}={\left[\begin{array}{ccc} \theta_{1d} & \theta_{2d} & \theta_{3d} \end{array}\right]}^{T}={\left[\begin{array}{ccc} 3 & -2 & 1 \end{array}\right]}^{T},\;\theta (0)={\left[\begin{array}{ccc} \theta_{10} & \theta_{20} & \theta_{30} \end{array}\right]}^{T}={\left[\begin{array}{ccc} 0 & 1 & -1 \end{array}\right]}^{T},\;\dot{\theta }(0)={\left[\begin{array}{ccc} {\dot{\theta }}_{10} & {\dot{\theta }}_{20} & {\dot{\theta }}_{30} \end{array}\right]}^{T}={\left[\begin{array}{ccc} 0 & 0 & 0 \end{array}\right]}^{T}.$$

The expression of the algorithm controller proposed in this paper is shown in Formula (27), and its detailed control parameters are as follows:

p=7, q=5, g=5, h=3, α=diag(5), β=diag(5), k=diag(5).

**Figure 6:**
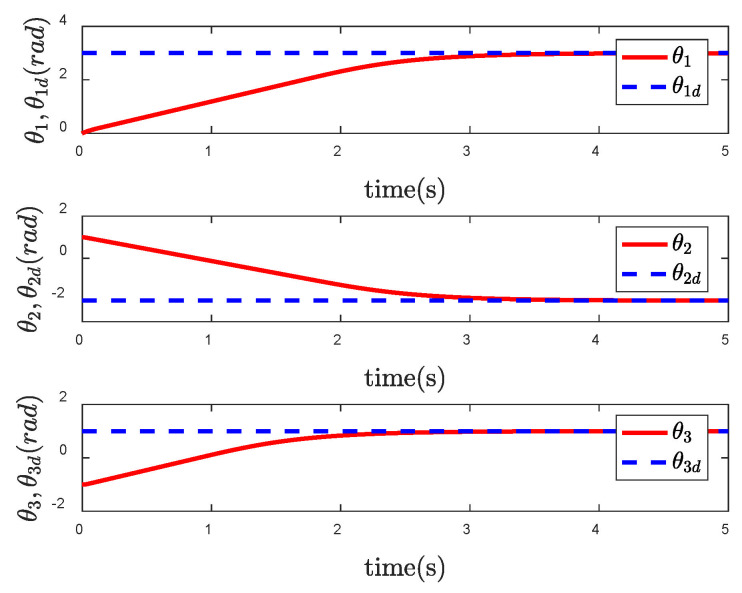
Tracking performance of θ under the SNFTSM controller.

**Figure 7:**
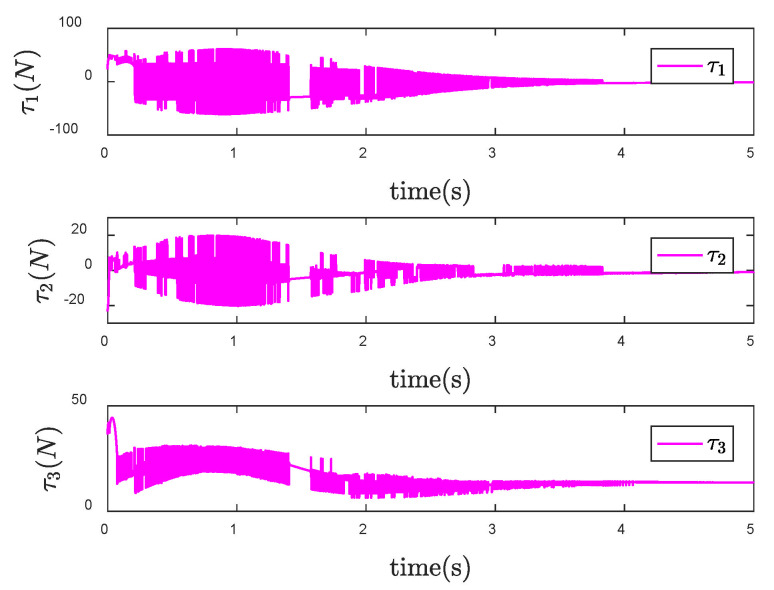
Control input τ of the SNFTSM controller when $\theta_{d}$ is a constant vector.

It is obvious from Figure 6 that the actual trajectory of each CFP joint can track to the desired trajectory in a short time, achieving the ideal control effect. However, from the control input torque of CFP system shown in Figure 7, it can be seen that due to the sliding mode control, there is a large degree of chattering, which is not conducive to the actual control.

In order to reduce the influence of chattering on the CFP system control, the boundary layer method combining signum function and saturation function is used to design the boundary layer function as follows:

$$\upsilon (s)=\left\{\begin{array}{l} \mathrm{sgn}(\frac{s}{\kappa }),\;\left|\frac{s}{\kappa }\right|>1\\ \frac{s}{\kappa },\;\;\;\;\;\;\left|\frac{s}{\kappa }\right|\leq 1 \end{array}\right.,$$

where κ is the size of the boundary layer.

**Figure 8:**
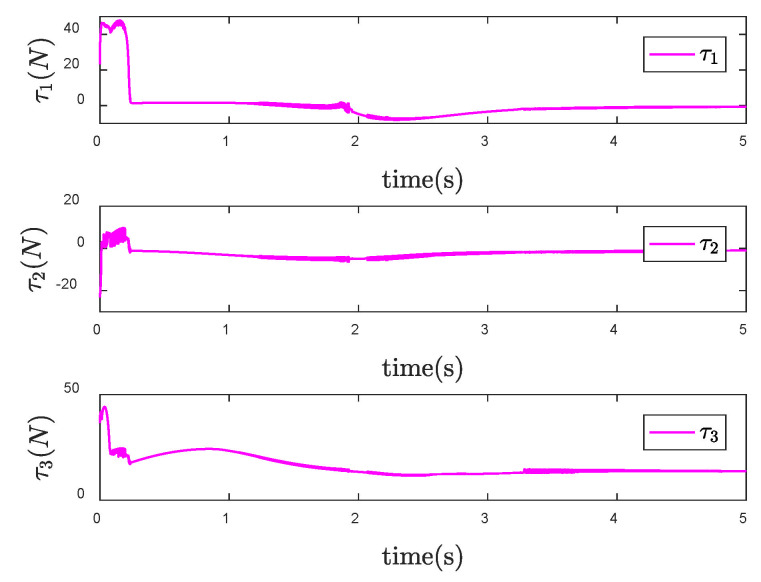
Control input τ of the SNFTSM controller with reduced chattering when $\theta_{d}$ is a constant vector.

Furthermore, the signum function in the original controller is replaced by the boundary layer function. Design the size of the boundary layer κ=0.1, and then the control input torque is shown in Figure 8. Compared with Figure 7, the chattering of the control torque is obviously weakened.

**Figure 9:**
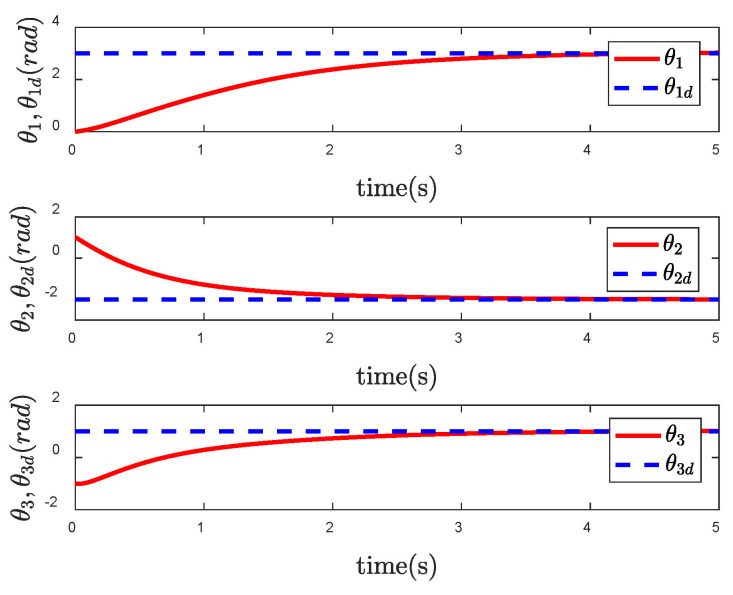
Tracking performance of θ under the NTSM controller.

**Figure 10:**
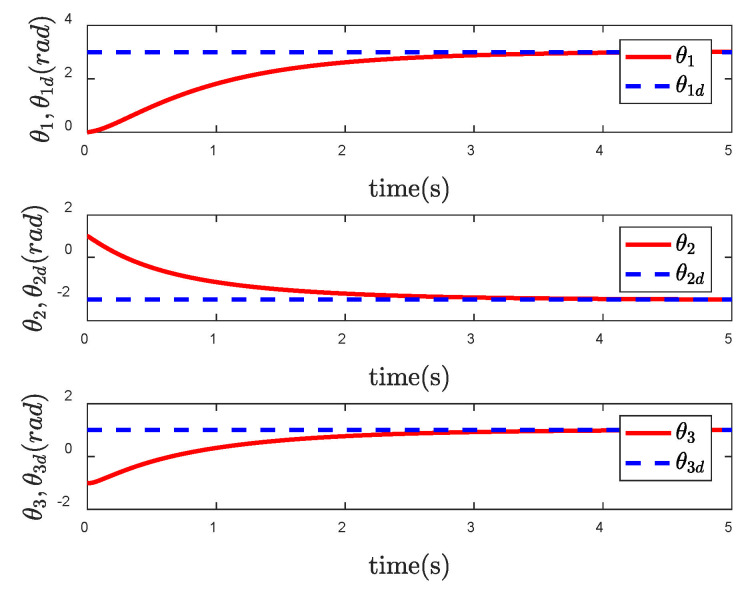
Tracking performance of θ under the NFTSM controller.

**Figure 11:**
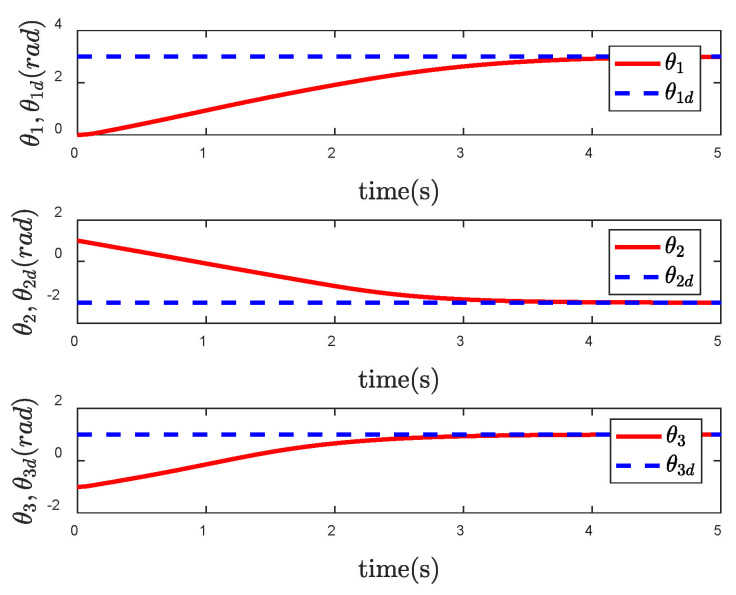
Tracking performance of θ under the SNTSM controller.

**Figure 12:**
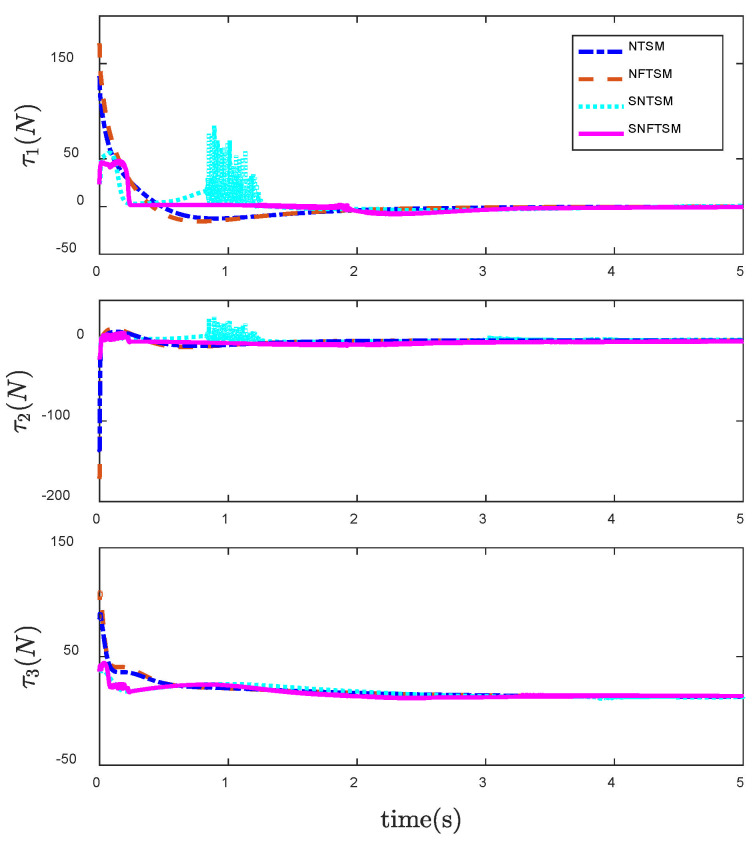
Comparison of control input τ under the NTSM, NFTSM, SNTSM and SNFTSM algorithms when $\theta_{d}$ is a constant vector.

In order to reflect the superiority of SNFTSM algorithm in the CFP tracking control field, we compare the simulation results with those of other sliding mode methods. To achieve a comparative effect, the controller parameter settings based on NTSM, NFTSM and SNTSM algorithms are consistent with SNFTSM algorithm. Figure 9, Figure 10 and Figure 11, respectively, depict the tracking curves of CFP joints driven by controllers designed based on NTSM, NFTSM and SNTSM algorithms. Figure 12 shows the comparison results of control torque of each algorithm.

However, only the general trend can be seen in figures, rather than specific data. According to Figure 6, Figure 7, Figure 8, Figure 9, Figure 10, Figure 11 and Figure 12, we can get simulation data in Table 1, where $t_{1}$, $t_{2}$ and $t_{3}$ represent the times when joint 1, joint 2 and joint 3 track the expected trajectory, respectively, and $\tau_{1max}$, $\tau_{2max}$ and $\tau_{3max}$ represent the maximum absolute value of each joint torque. It is obvious from the data in the table that the convergence time of the NFTSM algorithm is shorter than that of the NTSM algorithm. The reason is that the NFTSM algorithm increases the exponential term of error and improves the convergence speed of the algorithm. For the SNTSM algorithm, because it uses the saturation function, it can be seen from Table 1 that its convergence speed is not superior to the NTSM and NFTSM algorithms. Although the convergence speed of the SNFTSM algorithm proposed in this paper is also affected, compared with the SNTSM algorithm, it has a great improvement, and its convergence speed is only slightly lower than the NFTSM algorithm. However, it can be seen from the maximum joint torque of the system that the SNFTSM algorithm can greatly reduce joint torque while ensuring fast convergence. Compared with the NFTSM algorithm without a saturation function, the torque is reduced by at least 60%.

So as to further verify the control effect of the algorithm under the time-varying desired position, the desired trajectory and the initial state are designed as follows:

$$\theta_{d}={\left[\begin{array}{ccc} \sin(\pi t) & \cos(\pi t) & -\sin(\pi t) \end{array}\right]}^{T},{\dot{\theta }}_{d}={\left[\begin{array}{ccc} \pi \cos(\pi t) & -\pi \sin(\pi t) & -\pi \cos(\pi t) \end{array}\right]}^{T}.$$

Then, the initial state is designed as:

$$\theta (0)={\left[\begin{array}{ccc} 2 & -2 & 1 \end{array}\right]}^{T},\;\dot{\theta }(0)={\left[\begin{array}{ccc} 0 & -2 & -1 \end{array}\right]}^{T}.$$

**Figure 13:**
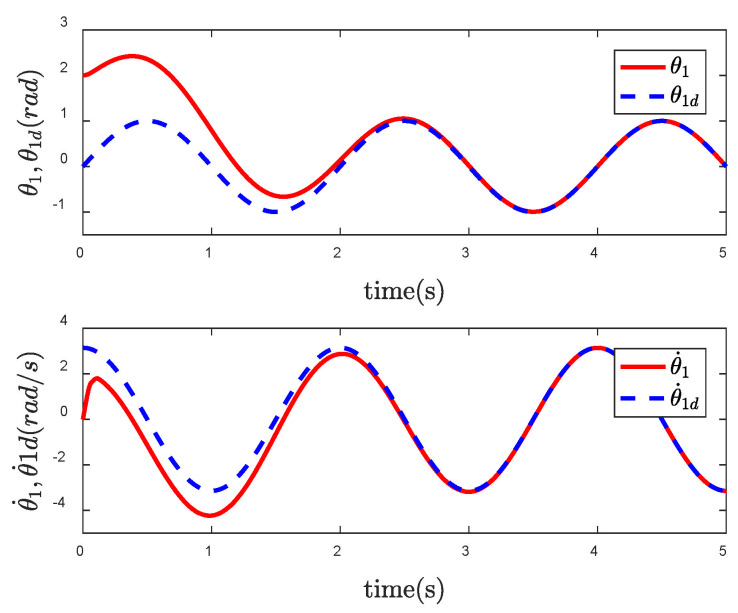
Tracking performance of joint 1.

**Figure 14:**
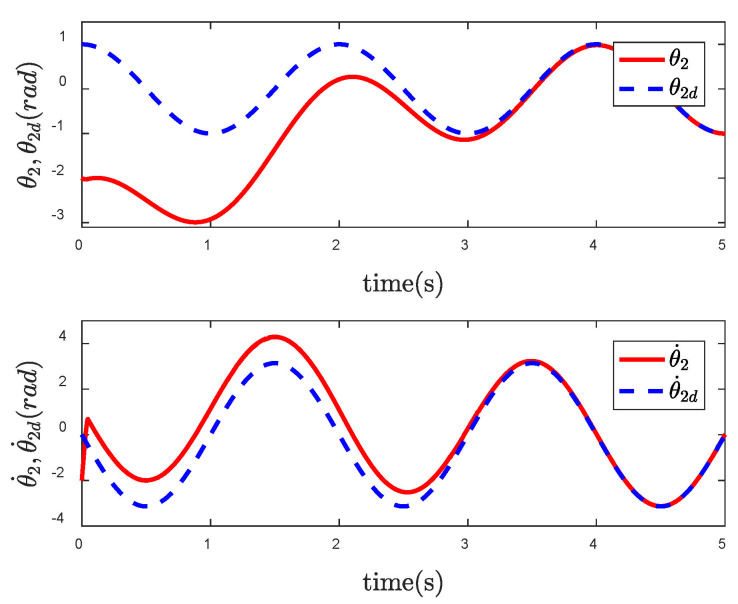
Tracking performance of joint 2.

**Figure 15:**
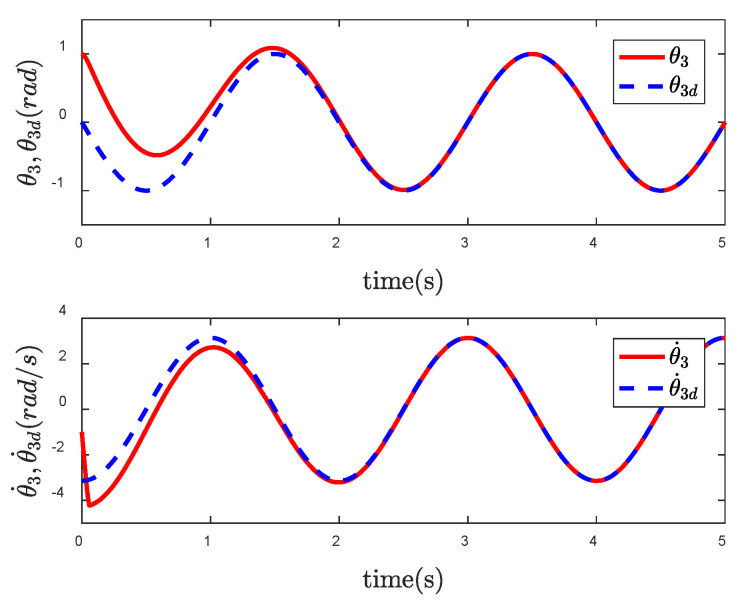
Tracking performance of joint 3.

**Figure 16:**
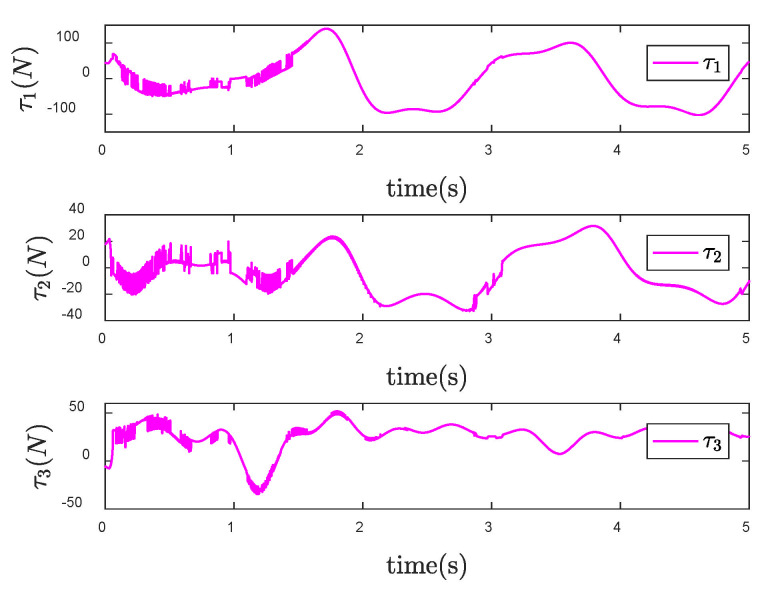
Control input τ of the SNFTSM controller when $\theta_{d}$ is a time-varying vector.

**Figure 17:**
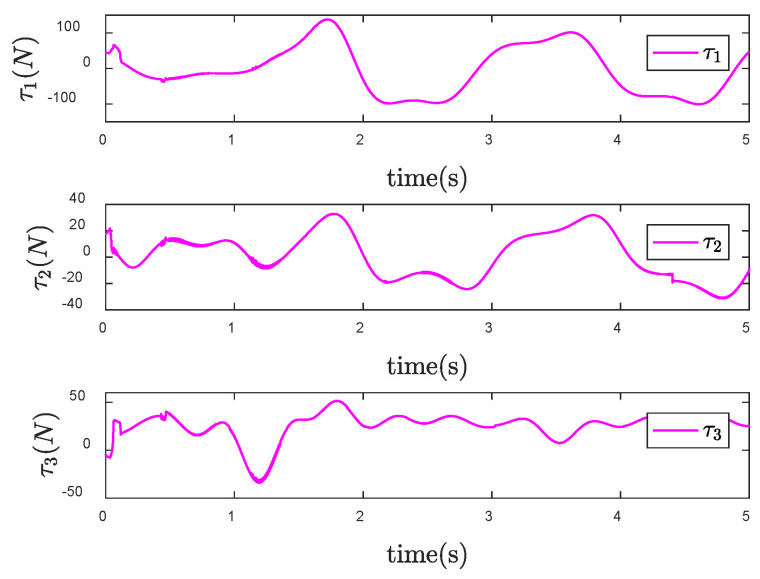
Control input τ of the SNFTSM controller with reduced chattering when $\theta_{d}$ is a time-varying vector.

**Figure 18:**
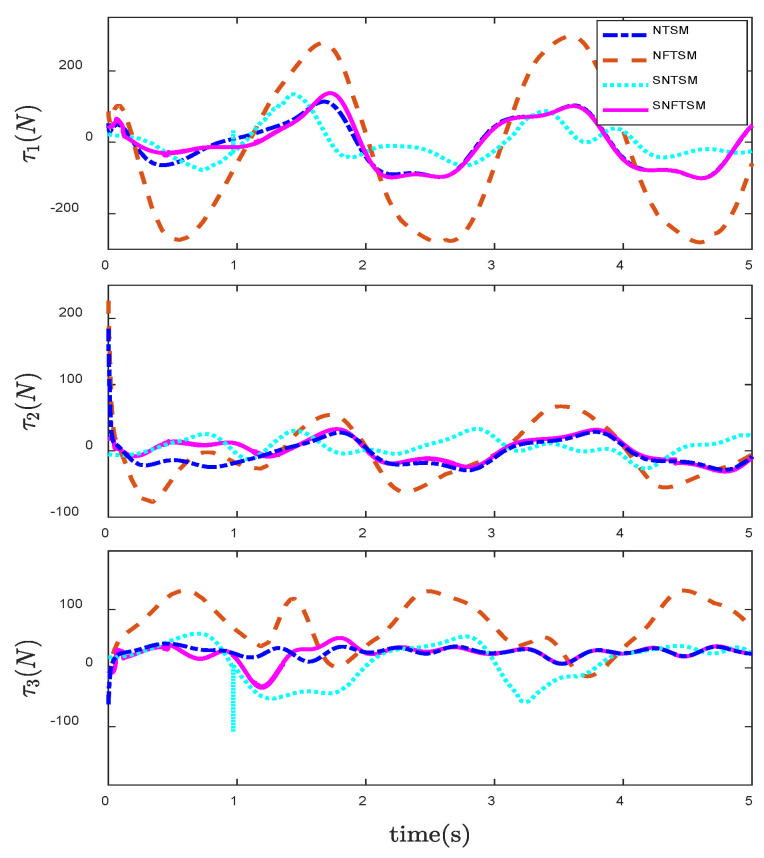
Comparison of control input τ under the NTSM, NFTSM, SNTSM and SNFTSM algorithms when $\theta_{d}$ is a time-varying vector.

Since the desired trajectory and desired speed are time-varying, only describing the position trajectory cannot reflect the tracking status of the system states. Figure 13, Figure 14 and Figure 15 show the position and speed tracking diagrams of each joint. It can be seen from the Figures that even if the desired trajectory is a time-varying curve, the actual state of the system can still track to the desired state in a short time. Figure 16 and Figure 17, respectively, describe the input torque of each CFP joint before and after the elimination of chattering. It can be clearly seen from the figure that after the reduction of chattering, the output of the controller is more stable and the fluctuation amplitude is greatly reduced. In Figure 18, the comparison of control torques of each algorithm is described, and it is obvious that the same conclusion can be drawn as in Figure 12, that is, the SNTSM algorithm and the SNFTSM algorithm can effectively reduce the amplitude of controller output, but the convergence speed of the SNFTSM algorithm is better than that of the SNTSM algorithm.

## 5. Conclusions

This paper combines the traditional NFTSM manifold with saturation functions and signum functions to design a novel sliding mode manifold. The new SNFTSM algorithm is used to design the trajectory tracking controller of CFP system. Its advantage is that the controller can effectively reduce the amplitude of the system input due to the existence of saturation functions, and it can ensure that the system state error converges to zero in a finite time. The disadvantage is that compared with the NFTSM controller, the convergence speed of the system state error will be slightly affected by saturation functions. However, compared with the large reduction of controller amplitude, the change of convergence time can be ignored. Finally, through simulation comparison, it is concluded that the SNFTSM algorithm can greatly reduce the amplitude of the controller input of the system while ensuring the convergence speed, achieving the expected design effect of the algorithm.

## Figures and Tables

**Table 1:** Simulation data.

	NTSM	NFTSM	SNTSM	SNFTSM
*t*1 *= s*	4.427	4.335	4.996	4.450
*t*2 *= s*	4.735	4.527	4.892	4.639
*t*3 *= s*	4.235	4.127	4.534	4.194
*t*1*max = N*	136.80	173.00	84.98	47.76
*t*2*max = N*	134.70	167.00	29.30	9.64
*t*3*max = N*	90.04	110.20	37.17	44.16

## Data Availability

All data in this paper are available upon request by contact with the corresponding author.
